# Clinical Significance of Endoscopic Improvement at 6 Months in Patients With Ulcerative Colitis Treated With Ustekinumab: A Retrospective Real‐world Analysis

**DOI:** 10.1002/deo2.70278

**Published:** 2026-01-20

**Authors:** Hiromu Morikubo, Minoru Matsuura, Haruka Komatsu, Takeshi Fujima, Ryota Ogihara, Noriaki Oguri, Tatsuya Mitsui, Daisuke Saito, Mari Hayashida, Jun Miyoshi, Tadakazu Hisamatsu

**Affiliations:** ^1^ Department of Gastroenterology and Hepatology Kyorin University School of Medicine Tokyo Japan

**Keywords:** clinical remission, endoscopic improvement, treat to target, ulcerative colitis, ustekinumab

## Abstract

**Objectives:**

Ustekinumab (UST), an anti‐interleukin‐12/23 p40 monoclonal antibody, has emerged as an effective therapeutic option for patients with moderate to severe ulcerative colitis (UC). However, early predictors of long‐term treatment response remain unclear. This study aimed to assess whether 6‐month endoscopic improvement (EI) predicts sustained clinical remission (CR) in patients with UC treated with UST.

**Methods:**

This was a retrospective observational study performed at Kyorin University Hospital. Patients with active UC (Lichtiger Index ≥ 5) who began UST between June 2020 and July 2023 were included. CR was assessed using the LI at weeks 4, 8, 16, and 24. EI at week 24 and sustained CR at week 56 were evaluated.

**Results:**

Fifty‐seven patients were enrolled, and the CR rate at week 24 was 57.9%. CR at week 4 was significantly associated with CR at week 24 (*p* = 0.004). Thirty‐one patients underwent colonoscopy at week 24. EI was achieved in 11 patients (35.5%), and patients with EI versus without EI at week 24 showed significantly higher rates of sustained CR at week 56 (90.0% sensitivity, 100.0% specificity; *p* = 0.005). The UST continuation rate was also significantly higher in the EI group compared with non‐EI patients (*p* = 0.04).

**Conclusions:**

EI 6 months after UST initiation was associated with sustained CR at week 56. This finding highlights the importance of early endoscopic assessment in optimizing long‐term outcomes in UST‐treated UC.

**Abbreviations**
CI: clinical improvement
CR: clinical remission
CS: colonoscopy
EI: endoscopic improvement
IL: interleukin
LI: Lichtiger index
MES: Mayo endoscopic subscore
NPV: negative predictive value
PPV: positive predictive value
UC: ulcerative colitis
UST: ustekinumab
VDZ: vedolizumab

## 1 Introduction

Ulcerative colitis (UC) is a chronic inflammatory bowel disease that significantly impairs patients’ quality of life [1]. While significant advancements have been made in UC management, with improved clinical outcomes [2], the development of effective therapies to achieve and maintain long‐term remission remains a clinical challenge. Ustekinumab (UST), a humanized monoclonal antibody targeting the p40 subunit of interleukin‐12 (IL‐12) and IL‐23, has emerged as a promising therapeutic option for patients with moderate to severe UC [3]. By inhibiting IL‐12 and IL‐23, UST suppresses the inflammatory response and promotes mucosal healing, thereby mitigating the symptoms of UC [4]. This mechanism of action has been demonstrated in a large phase 3 clinical trial [3], which showed that UST was significantly more effective than placebo in inducing and maintaining clinical remission (CR) in patients with UC. However, the efficacy of these molecular targeted therapies can vary widely among individuals. Data from clinical trials involving patients with well‐defined backgrounds are likely to differ from real‐world clinical data, and it is difficult to predict treatment response prior to initiation on the basis of clinical trial data alone. Therefore, identifying early biomarkers that can predict long‐term outcomes is crucial to optimizing patient care.

This study aimed to investigate whether endoscopic improvement (EI) after 6 months of UST treatment is a predictive biomarker for sustained CR in patients with UC. By identifying patients who are likely to respond well to UST early in the treatment course, clinicians can tailor treatment strategies and improve patient outcomes.

## 2 Methods

### 2.1 Study Setting

This single‐center retrospective observational study was performed at our hospital. Patients with UC who began UST as induction therapy (Lichtiger index (LI) ≥ 5) between June 2020 and July 2023 were included. Patients who underwent colonoscopy (CS) 24 weeks after UST initiation were followed up to 56 weeks.

### 2.2 Data Collection

The collected data comprised the patients’ demographic characteristics (age, sex, disease duration, disease type, prior history of UC treatment, and concomitant medications), clinical disease activity data (e.g., LI and Mayo endoscopic subscore [MES]), and laboratory data (e.g., C‐reactive protein, albumin, and hemoglobin) at baseline. The LI was recorded at each visit to the hospital (week 4, 8, 16, and 24).

### 2.3 Outcomes and Definition

The primary outcome was the correlation between EI at week 24 and sustained CR at week 56. The secondary outcomes were the clinical improvement (CI) rate, CR rate, and endoscopic remission rate at week 24; CR rate and UST continuation rate at week 56; and the association between baseline patient characteristics and EI.

CI was defined as an LI ≤ 10 and an LI decrease of > 3 points from the initiation of UST therapy. CR was defined as an LI ≤ 3. Furthermore, if CR was achieved at both week 24 and week 56, this was defined as sustained CR. EI and endoscopic remission (ER) were defined as an MES of ≤ 1 and 0, respectively.

### 2.4 Statistical Analysis

All statistical analyses were performed using STATA/S v. 15.1 (Stata Corporation, College Station, TX, USA). Continuous variables are expressed as median and interquartile range or mean ± standard deviation. Categorical variables are expressed as numbers and percentages (%). Missing values at baseline were not imputed, and if a scheduled visit was missed, clinical activity was assumed to be the same as that at the previous visit. The Mann–Whitney U test was used to compare continuous variables, and Fisher's exact test was used to compare categorical variables. Sensitivity, specificity, positive predictive value (PPV), and negative predictive value (NPV) were reported with 95% confidence intervals in brackets. The association of CR at week 24 with CR at weeks 4, 8, and 16 was evaluated using Fisher's exact test. Subsequently, we performed a univariate and multivariate logistic regression analysis adjusting for other clinical factors, including normalization of CRP and albumin. Drug continuation rates were assessed using Kaplan–Meier curves, and the log‐rank test was used to compare the differences between groups.

## 3 Results

### 3.1 Study Overview and the Real‐World Outcomes of UST Therapy

**FIGURE 1:**
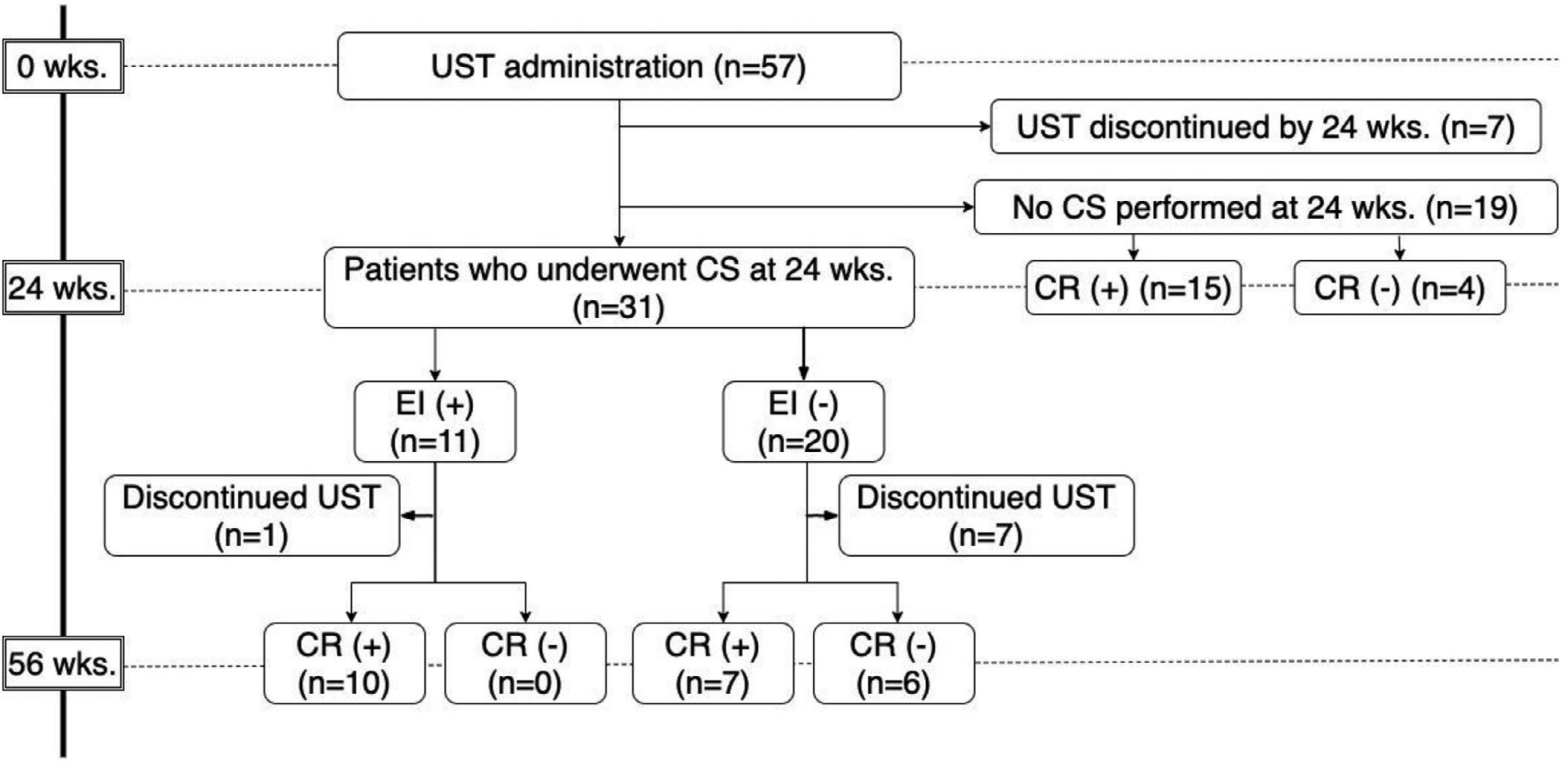
Study overview. Flowchart illustrating the study design and patient disposition. Patients with ulcerative colitis who initiated ustekinumab were followed for up to 56 weeks. The diagram shows the number of patients who underwent colonoscopy at week 24 and were stratified by endoscopic improvement. EI: endoscopic improvement, CS: colonoscopy, CR: clinical remission, UST: ustekinumab.

**FIGURE 2:**
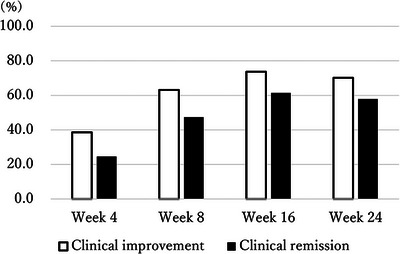
Clinical improvement and clinical remission rates up to week 24. Clinical improvement (CI) and clinical remission (CR) rates at weeks 4, 8, 16, and 24 after the initiation of UST. Data are presented as percentages of patients achieving CI and CR at each time point (*N* = 57).

A study overview is shown in Figure 1. Fifty‐seven patients were enrolled, 7 of whom discontinued UST by week 24 due to primary non‐response, and 31 underwent CS 24 weeks from the initiation of UST. Nineteen patients did not undergo CS at week 24, and only the clinical score was evaluated. The patients’ baseline characteristics are shown in Table 1. Thirty‐two patients were male (56.1%), the median age was 36.2 (27.7–53.3) years, and the median disease duration was 6.5 (2.9–10.6) years. Steroid‐dependent and refractory patients comprised 33 (57.9%) and 19 patients (33.3%), respectively. Fifteen patients were biologics‐naïve (26.3%). The median LI and MES were 8 (6–10) and 3 (2–3), and the patients had moderate to severe UC. In this cohort, corticosteroids and tacrolimus, listed in Table 1, were discontinued after the initiation of UST, and no patients continued these medications up to week 24. Only three patients received UST at 12‐week intervals. As shown in Figure 2, the CI and CR rates over time were as follows: At week 4, 38.6% of the patients achieved CI, while 24.6% achieved CR. By week 8, these rates increased to 63.2% for CI and 47.4% for CR. At week 16, 73.7% of the patients had achieved CI, and 61.4% had achieved CR. By week 24, the rates were 70.2% for CI and 57.9% for CR. Of 31 patients who received CS at week 24, 11 patients (35.5%) achieved EI; 20 patients did not. The ER rate was 19.4% (6 patients).

**TABLE 1:** Patients' characteristics.

	Baseline (*N* = 57)	Patients who received CS at 24 weeks (*N* = 31)
Male, *n* (%)	32 (56.1)	17 (54.8)
Age (median IQR, years)	36.2 (27.7–53.3)	36.1 (27.7–49.1)
Smoking (current/past/never), *n* (%)	5/7/45 (9/12.3/78.8)	2/3/26 (6.5/9.7/83.8)
Age of onset (median IQR, years)	24 (19–46)	25 (20–44)
Disease duration (median IQR, years)	6.5 (2.9–10.6)	6.6 (3.8–10.6)
Disease extent (pancolitis/left‐sided), *n* (%)	40/17 (70.1/29.9)	22/9 (71.0/29.0)
Steroid (naïve/dependent/refractory), *n* (%)	5/33/19 (8.8/57.9/33.3)	3/19/9 (9.6/61.4/29.0)
Biologics naïve, *n* (%)	15 (26.3)	9 (29.0)
Concomitant medication		
5‐ASA, *n* (%)	42 (73.7)	25 (80.6)
Immunomodulator, *n* (%)	14 (24.6)	10 (32.3)
Prednisolone, *n* (%)	6 (10.5)	4 (12.9)
Tacrolimus, *n* (%)	2 (3.5)	1 (3.2)
Lichtiger index (median IQR)	8 (6–10)	8 (6–10)
Mayo endoscopic subscore (median IQR)	3 (2–3)	2 (2–3)
Hb (median IQR, g/dL)	12.3 (10.9–13.8)	12.7 (11.2–14.0)
Alb (median IQR, g/dL)	3.6 (3.5–4.0)	3.7 (3.5–3.9)
CRP (median IQR, mg/dL)	0.39 (0.11–1.84)	0.30 (0.07–1.18)

Abbreviations: 5‐ASA, 5‐aminosalicylic acid; Alb, albumin; CRP, C‐reactive protein; CS, colonoscopy; Hb, hemoglobin; IQR, interquartile range.

Of the patients who achieved EI at week 24, 1 discontinued UST because of a UC‐associated neoplasm, and 10 achieved CR at week 56. Of the patients who did not achieve EI at week 24, seven discontinued UST, seven achieved CR at week 56, and six did not. Among patients not undergoing CS, 15 achieved CR at week 24, and 4 did not (Figure 1).

Only one patient discontinued UST because of a drug eruption, which improved after UST discontinuation.

### 3.2 Factors Associated With Clinical or Endoscopic Outcomes at Week 24

First, the patients who achieved CR at week 24 were assessed. The patients who did not achieve CR at week 24 had significantly higher baseline LI compared with those who achieved (*p* = 0.029) (Table S1). In contrast, the achievement of CR at week 4 after the initiation of UST was statistically significantly associated with CR at week 24 (sensitivity: 39.4% [22.9–57.9], specificity: 95.8% [78.9–99.9], PPV: 92.9% [66.1–99.8], NPV: 53.5% [37.7–68.8]; *p* = 0.004) (Table 2), as was the achievement of CR in subsequent weeks (both at week 8 and 16; *p* < 0.001) (Tables S2 and S3). In the multivariate analysis, week‐4 CR remained an independent predictor of CR at week 24 (adjusted odds ratio 15.29 [1.77–131.90], *p* = 0.013) (Table 3).

**TABLE 2:** Clinical remission week 4 for estimating clinical remission week 24.

		Clinical remission at week 24	
		(+)	(‐)
Clinical remission at week 4	(+)	13	1
	(‐)	20	23

**TABLE 3:** Multivariate predictors at week 4 for clinical remission at week 24.

Variable	Univariate OR (95% CI)	*p*‐Value	Adjusted OR (95% CI)	Adjusted *p‐*Value
CR at week 4	14.95 (1.79–124.60)	0.012	15.29 (1.77–131.90)	0.013
CRP normalized at wk4	1.39 (0.45–4.30)	0.570	0.93 (0.26–3.37)	0.915
Alb normalized at wk4	1.77 (0.61–5.17)	0.296	1.80 (0.56–5.80)	0.326

Hosmer–Lemeshow *χ* ^2^ = 2.20, *p* = 0.8206. Pseudo *R* ^2^ = 0.154.

Second, the patients who had undergone CS 24 weeks from the initiation of UST were divided into EI achievement and non‐achievement groups. No statistically significant baseline characteristics were associated with achieving EI, including age, gender, disease type, disease severity, and biologics‐naïve status or biologics‐experienced status (Table 4). Furthermore, CI and CR at each assessment point were not statistically significantly associated with EI.

**TABLE 4:** Univariate analysis of the association of background factors for endoscopic improvement at 24 weeks.

*N* = 31	Endoscopic remission (+), (*N* = 11)	Endoscopic remission (‐), (*N* = 20)	*p*‐Value
Male, *n* (%)	6 (54.5)	11 (55.0)	1.000^b^
Age (median IQR, years)	38.9 (27.5–48.5)	33.1 (28.1–51.2)	0.563*
Age of onset (median IQR, years)	25 (20–44)	24 (20–42)	0.756*
Smoking (current/past/never), *n* (%)	1/1/9 (9.1/9.1/81.8)	1/2/17 (5.0/10.0/85.0)	1.000^b^
Disease duration (median IQR, years)	7.2 (4.4–13.6)	6.6 (3.8–8.7)	0.496*
Disease extent (pancolitis/left‐sided), *n* (%)	9/2 (81.8/18.2)	13/7 (65.0/35.0)	0.429^b^
Steroid (naïve/dependent/refractory), *n* (%)	2/8/1 (18.2/72.7/9.1)	1/11/8 (5.0/55.0/40.0)	0.190^b^
Biologics naïve, *n* (%)	2 (18.2)	6 (30.0)	0.676^b^
Lichtiger index (median IQR)	7 (5–10)	8 (7–11)	0.289*
Mayo endoscopic subscore (median IQR)	2 (2–3)	2 (2–3)	0.156*
Hb (median IQR, g/dL)	12.8 (11.2–14.3)	12.0 (11.0–13.9)	0.549*
Alb (median IQR, g/dL)	3.7 (3.5–3.9)	3.7 (3.5–4.1)	0.931*
CRP (median IQR, mg/dL)	0.33 (0.06–2.29)	0.21 (0.07–0.89)	0.366*

^a^ Mann‐Whitney U test.

^b^ Fisher's exact test.

Alb, albumin; CRP, C‐reactive protein; Hb, hemoglobin; IQR, interquartile range.

### 3.3 Association Between Sustained CR at Week 56 and Endoscopic Improvement at Week 24

**FIGURE 3:**
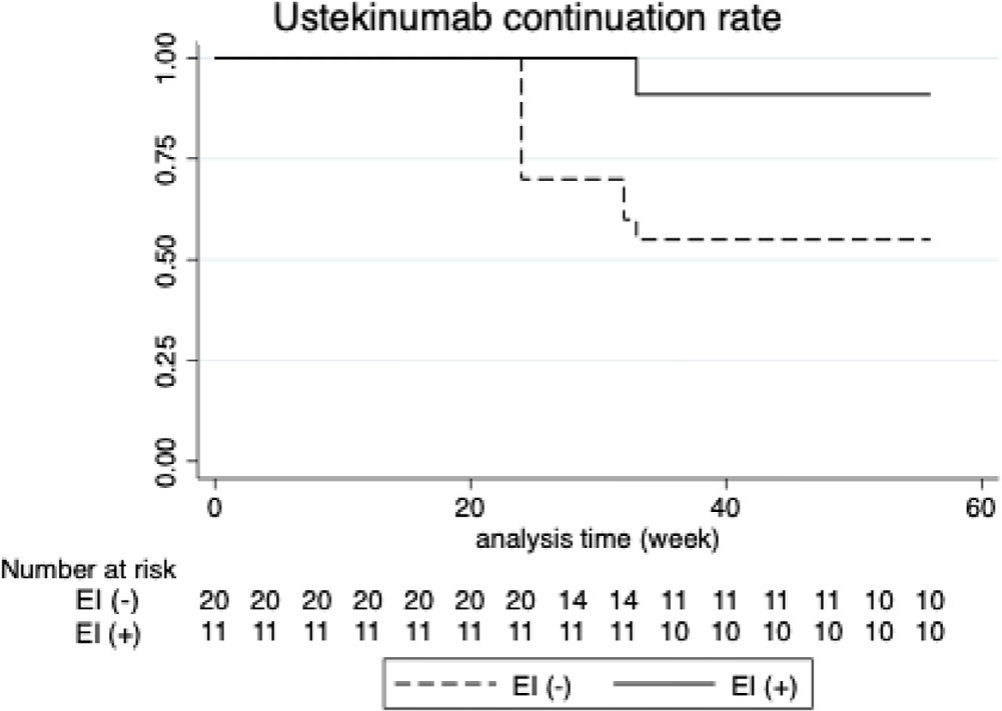
Ustekinumab continuation rate in patients undergoing colonoscopy at week 24. Kaplan–Meier survival curve showing the drug continuation rate of ustekinumab after colonoscopy at week 24. The comparison between patients with and without endoscopic improvement was evaluated using the log‐rank test (*p* = 0.04). EI: endoscopic improvement.

**FIGURE 4:**
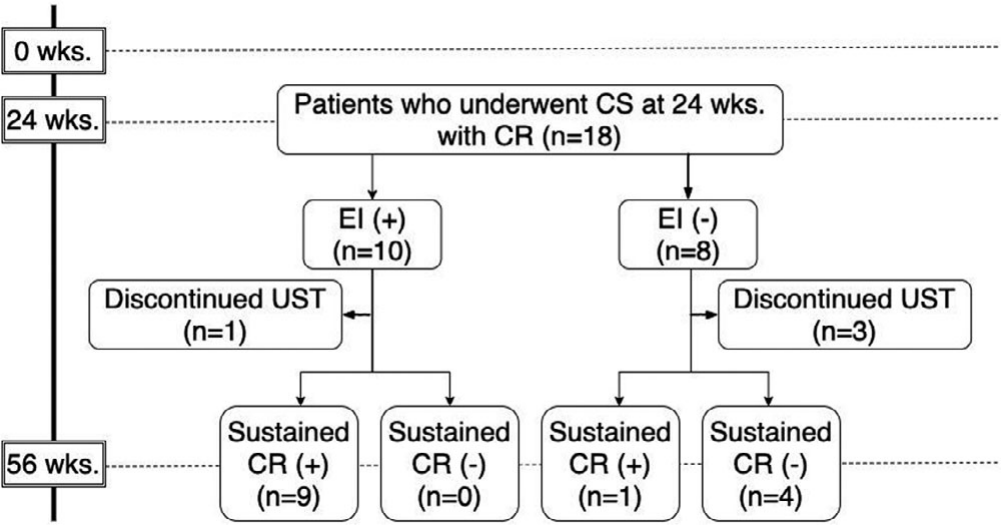
Study overview of the patients undergoing colonoscopy and in clinical remission at week 24. Patients who achieved clinical remission at week 24 and underwent colonoscopy were stratified by the presence or absence of endoscopic improvement. EI: endoscopic improvement, CS: colonoscopy, CR: clinical remission, UST: ustekinumab.

The Kaplan–Meier curve in Figure 3 shows the UST continuation rate in patients who underwent CS at week 24. Compared with the group that achieved EI at CS, the UST continuation rate was statistically significantly lower in the group that did not achieve EI (log rank test, *p* = 0.04). In particular, discontinuation of UST after 20 weeks was observed in the EI non‐achievers. Next, to evaluate the significance of achieving EI at week 24, the patients who achieved CR at week 24 were examined separately in the EI‐achieved group (CR+EI+ group) and the EI‐unachieved group (CR+EI− group) (Figure 4). Eighteen patients achieved CR at week 24 and also underwent CS, 10 in the CR+EI+ group and 8 in the CR+EI− group. Only one patient in the CR+EI+ group discontinued UST because of a neoplasm; all others achieved sustained CR, even at week 56. In contrast, three patients in the CR+EI− group discontinued UST because of the UC disease course, and four patients had worsened symptoms at week 56 and were no longer in CR. Only one patient achieved sustained CR up to week 56.

The results of the analysis of the association between EI at week 24 and sustained CR at week 56 are shown in a 2×2 table (Table 5). There was a significant association between EI at week 24 and sustained CR at week 56 (*p* = 0.005), with EI at week 24 having a sensitivity of 90.0% [55.5–99.7], specificity of 100.0% [39.8‐100.0], PPV of 100.0% [66.4–100.0], and NPV of 80.0% [28.4–99.5], and achieving EI at week 24 predicted sustained CR at week 56. There was no significant association between Hb (*p* = 0.887), Alb (*p* = 0.435), or CRP levels (*p* = 0.670) at week 24 and sustained CR at week 56.

**TABLE 5:** Endoscopic improvement at week 24 for estimating sustained clinical remission.

		Sustained clinical remission	
		(+)	(‐)
**Endoscopic improvement at week 24**	(+)	9	0
	(‐)	1	4

## 4 Discussion

This retrospective observational study was performed to evaluate real‐world data on UST treatment for UC and to identify early predictors of therapeutic efficacy. The results showed that CR at week 4 was significantly associated with CR at week 24. Furthermore, EI at week 24 was associated with sustained CR at week 56 and with treatment persistence, highlighting its value as a predictive marker for long‐term outcomes.

Although therapeutic options for UC have improved [5] and the colectomy rate has decreased [2], accurate evaluation of treatment and maintenance of long‐term remission is still very important. A phase 3 clinical trial of UST [3] and subsequently published real‐world data [6] did not report any clear factors prior to the initiation of UST that predict the efficacy of UST. Hong et al. reported that anti‐tumor necrosis factor drug non‐responders and MES 3 were negative predictors of CR 3 months after induction, but these factors were not significant 12 months after induction [7]. Omori et al. [8] reported that gender and body mass index before the initiation of UST were associated with CR at 8 weeks, but it is difficult to predict mid‐ to long‐term outcomes at 1 year. Similarly, our study did not identify significant predictors among the baseline characteristics, underscoring the need for on‐treatment evaluation to guide long‐term management.

Notably, despite the high rate of biologics‐failure cases (70%) in this study, the efficacy rate of UST was comparable to that in a previous study [9], showing that biologics failure was not associated with CR and EI at week 24 in the univariate analysis. The efficacy of vedolizumab (VDZ) treatment is lower in patients with UC who have been treated with one or more biologic agents than in biologics‐naïve patients [10]. In contrast, Fumery et al. [11] reported higher efficacy with UST compared with VDZ in patients who had received one or more biologic agent. Meta‐analysis also demonstrated efficacy after changing from anti‐tumor necrosis factor‐alpha antibody agents to UST [12]. These findings suggest that, unlike VDZ, UST retains therapeutic potential in biologic‐experienced populations, supporting its use in refractory UC.

Importantly, this study showed that CR at week 4 can predict CR at week 24, whereas CRP or albumin did not. Assessing treatment efficacy at such an early time point is particularly valuable, given the challenges in predicting individual response to UST prior to initiation of therapy. While previous studies have reported that the clinical response at 8 weeks was associated with CR at 56 weeks [8, 9], our findings suggest that treatment response can be evaluated even earlier. Chen et al. showed that CR at week 2 was associated with favorable outcomes in the clinical trial [13]. In our real‐world cohort, week‐2 data were not systematically collected. Nevertheless, the findings from the post‐hoc analysis are consistent with our results. Importantly, our research reinforces the importance of early treatment response in real‐world clinical practice, consistent with findings from clinical trials. Early identification of non‐responders could facilitate timely therapeutic adjustments, thus potentially improving outcomes.

Recently, the importance of targeting EI has been increasingly emphasized in therapeutic strategies for UC. The STRIDE‐II consensus particularly recommends achieving EI, in addition to CR, as a treatment goal for UC [14]. The present study demonstrated that EI at week 24 following UST initiation was predictive of both subsequent treatment persistence and sustained CR at week 56. These findings support the evidence for the validity of EI as a therapeutic target in the management of UC with UST. Previous clinical trial data have demonstrated that patients who successfully transitioned from induction to maintenance therapy with UST maintained favorable long‐term outcomes for up to 4 years [15, 16]. Although the present study did not assess long‐term outcomes, the finding that EI at week 24 predicted sustained CR suggests the potential of this finding as a surrogate marker for long‐term therapeutic success. However, the high sensitivity and specificity should be interpreted with caution, as the small sample size results in a wide 95% confidence interval. This highlights the clinical importance of the 24‐week endoscopic evaluation, which can be a valuable tool to predict the long‐term efficacy of UST in patients with UC.

Notably, approximately 35% of the patients without EI at week 24 eventually achieved CR by week 56, indicating that a delayed response to UST may occur. Compared with UST early responders, delayed responders had higher MES values, indicating more severe disease, at baseline, and comparable 1‐year outcomes for early and delayed responders [17]. Therefore, while EI at week 24 is useful, caution is needed when interpreting this finding as a definitive indication for treatment change, and future prospective intervention studies are warranted.

Regarding the safety profile of UST, Miyoshi et al. highlighted its favorable safety characteristics [18]. In the present cohort, one patient discontinued UST because of a UST‐induced drug eruption. Previous case reports have demonstrated that UST‐induced drug eruptions typically resolve promptly following drug withdrawal [19], and a similar clinical course was observed in the patient in our study. Additionally, one patient in this cohort underwent colectomy because of dysplasia, prompting cessation of UST. Notably, long‐term data from the UNIFI study have not reported an increased incidence of malignancy associated with UST use [15]. Furthermore, Hasan et al. [20] indicated that UST does not elevate the risk of cancer recurrence in patients with a prior history of malignancy. These findings support the favorable safety profile of UST in real‐world settings, which was also affirmed in the present study.

This study has several limitations. First, CS at week 24 was not performed for all patients, resulting in a small sample size. The inability to perform endoscopic evaluation for all patients within the time period defined in a study remains a challenge for future studies. However, the endoscopic evaluation at week 24 in this study was performed as part of daily practice and provides valuable real‐world insights that are different from those obtained in clinical trials. Second, we did not evaluate the efficacy of UST dosing every 8 weeks versus every 12 weeks in detail. Although the data from the UNIFI trial suggest no clear difference in CR rates between these dosing intervals [15], previous studies have reported that serum UST concentrations may be associated with endoscopic and histological activity [21] and that dose escalation may be beneficial in certain patients [22]. Given that only three patients in our cohort received the drug every 12 weeks, and the majority were treated with the 8‐week regimen, further investigation is needed to clarify optimal dosing strategies. Third, the retrospective study design limits the ability to infer causality. Future prospective studies are warranted to validate the association between early clinical response at week 4 and clinical and EI at week 24. In particular, it is necessary to assess whether early treatment adjustments—such as dose intensification or changing to an alternate therapy—will improve long‐term outcomes in patients with an inadequate early response. Additionally, standardization of endoscopic assessment at week 24 and evaluation of its impact on long‐term prognosis is important. Although this study assessed outcomes up to 56 weeks, future studies should aim to determine the long‐term sustainability of EI and its association with clinically meaningful endpoints, such as surgery‐free survival, steroid dependency, and hospitalization rates. Fecal calprotectin and leucine‐rich alpha 2 glycoprotein were not included in the analysis due to limited real‐world measurement and numerous missing values.

In conclusion, our study suggests that early CR at week 4 and EI at week 24 are important indicators of mid‐ to long‐term treatment success with UST in UC. These findings provide practical guidance for early therapeutic assessment and support the finding that UST is an effective treatment option, even for patients with prior biologics failure.

## Author Contributions

**Conceptualization**: Hiromu Morikubo and Minoru Matsuura. **Methodology**: Hiromu Morikubo, Minoru Matsuura, Jun Miyoshi, and Tadakazu Hisamatsu. **Formal analysis**: Hiromu Morikubo and Minoru Matsuura. **Investigation**: Hiromu Morikubo and Takeshi Fujima. **Resources**: Hiromu Morikubo, Minoru Matsuura, Haruka Komatsu, Takeshi Fujima, Ryota Ogihara, Noriaki Oguri, Tatsuya Mitsui, Daisuke Saito, Mari Hayashida, Jun Miyoshi, and Tadakazu Hisamatsu. **Data Curation**: Hiromu Morikubo and Minoru Matsuura. **Writing – Original Draft**: Hiromu Morikubo. **Writing – Review & Editing**: All authors. **Visualization**: Hiromu Morikubo, Minoru Matsuura, Takeshi Fujima, Ryota Ogihara, Jun Miyoshi, and Tadakazu Hisamatsu. **Supervision**: Tadakazu Hisamatsu. **Project administration**: Minoru Matsuura. **Funding acquisition**: Hisamatsu Tadakazu.

## Funding

This work was supported in part by grants from the Japan Science Research Grant for Research on Intractable Diseases (Japanese Inflammatory Bowel Disease Research Group) affiliated with the Japan Ministry of Health, Labor and Welfare.

## Ethics Statement

This study was performed in accordance with the guidelines of the Declaration of Helsinki and was approved by the Institutional Ethics Committees of Kyorin University School of Medicine (Approval Number: 1378). This study used previously recorded data, and the ethics committee waived the need to obtain informed consent.

## Conflicts of Interest

Hiromu Morikubo has received grant support from Takeda Pharmaceutical and consulting and lecture fees from AbbVie GK, JIMRO Co., Ltd., and Mochida Pharmaceutical Co., Ltd. Minoru Matsuura has received consulting and lecture fees from Janssen Pharmaceutical K.K., Takeda Pharmaceutical Co., Ltd., AbbVie GK, Mitsubishi Tanabe Pharma Corp., Kyorin Pharmaceutical Co., Ltd., Mochida Pharmaceutical Co., Ltd., JIMRO Co., Nippon Kayaku Co., Ltd., Mylan EPD G.K., and Aspen Japan Co., Ltd. Daisuke Saito has received lecture fees from Mitsubishi Tanabe Pharma Corporation, AbbVie GK, EA Pharma Co., Ltd., Kyorin Pharmaceutical Co., Ltd., JIMRO Co., Janssen Pharmaceutical K.K., Mochida Pharmaceutical Co., Ltd., Takeda Pharmaceutical Co., Ltd., and Pfizer Inc. Jun Miyoshi has received lecture fees from EA Pharma Co., Ltd., AbbVie GK, Janssen Pharmaceutical K.K., Pfizer Inc., Mitsubishi Tanabe Pharma Corporation, JIMRO Co., Miyarisan Co., Ltd., Nippon Kayaku Co., Ltd., Mochida Pharmaceutical Co., Ltd., and Takeda Pharmaceutical Co., Ltd. Tadakazu Hisamatsu has performed joint research with EA Pharma Co., Ltd. and Kissei Pharmaceutical Co., Ltd. Tadakazu Hisamatsu has received grant support from Mitsubishi Tanabe Pharma Corp., EA Pharma Co., Ltd., AbbVie GK, JIMRO Co., Ltd., Zeria Pharmaceutical Co., Ltd., Kyorin Pharmaceutical Co., Ltd., Nippon Kayaku Co., Ltd., Takeda Pharmaceutical Co., Ltd., Pfizer Inc., Boston Scientific Co., Ltd., and Mochida Pharmaceutical Co., Ltd. Tadakazu Hisamatsu has also received consulting and lecture fees from EA Pharma Co., Ltd., AbbVie GK, Janssen Pharmaceutical K.K., Pfizer Inc., Mitsubishi Tanabe Pharma Corporation, Kyorin Pharmaceutical Co., Ltd., JIMRO Co., Mochida Pharmaceutical Co., Ltd., Gilead Sciences Inc., Bristol Myers Squibb Co., Ltd., Eli Lilly, Abivax, and Takeda Pharmaceutical Co., Ltd. Haruka Komatsu, Takeshi Fujima, Ryota Ogihara, Noriaki Oguri, Tatsuya Mitsui, and Mari Hayashida declare no conflicts of interest.

## Supporting information




**Supporting Table 1**: Univariate analysis of the association of background factors for CR at week 24. Univariate analysis of baseline characteristics associated with clinical remission at week 24 in patients with ulcerative colitis treated with ustekinumab (*N* = 50). Data are presented as numbers (%) or medians (interquartile range, IQR). *Mann‐Whitney U test; §Fisher's exact test. Abbreviations: Alb, albumin; CRP, C‐reactive protein; Hb, hemoglobin; IQR, interquartile range.
**Supporting Table 2**: Clinical improvement week 8 for estimating endoscopic remission. Association between clinical remission (CR) at week 8 and CR at week 24 in patients treated with ustekinumab (*p* < 0.001).
**Supporting Table 3**: Clinical improvement week 16 for estimating endoscopic remission. Association between CR at week 16 and CR at week 24 in patients treated with ustekinumab (*p* < 0.001).

## Data Availability

The data underlying this article will be shared by the corresponding author upon reasonable request.
