# Supplementary material for: Clinical Significance of Endoscopic Improvement at 6 Months in Patients With Ulcerative Colitis Treated With Ustekinumab: A Retrospective Real‐world Analysis
Source: DEN Open. 2026 Jan 20;6(1):e70278. doi: 10.1002/deo2.70278 (PMC12817195; doi:10.1002/deo2.70278)
Supplement: Supplementary file 1 — Supporting Table 1: Univariate analysis of the association of background factors for CR at week 24. Univariate analysis of baseline characteristics associated with clinical remission at week 24 in patients with ulcerative colitis treated with ustekinumab (N = 50). Data are presented as numbers (%) or medians (interquartile range, IQR). *Mann‐Whitney U test; §Fisher's exact test. Abbreviations: Alb, albumin; CRP, C‐reactive protein; Hb, hemoglobin; IQR, interquartile range. Supporting Table 2: Clinical improvement week 8 for estimating endoscopic remission. Association between clinical remission (CR) at week 8 and CR at week 24 in patients treated with ustekinumab (p < 0.001). Supporting Table 3: Clinical improvement week 16 for estimating endoscopic remission. Association between CR at week 16 and CR at week 24 in patients treated with ustekinumab (p < 0.001). [file DEO2-6-e70278-s001.docx]

**Supplementary Tables**

Supplementary table 1. Univariate analysis of the association of background factors for clinical remission at 24 weeks.

|  | Clinical remission (+), (N=33) | Clinical remission (-), (N=24) | *P* value |
| --- | --- | --- | --- |
| Male, n (%) | 18 (54.5) | 14 (58.3) | 0.794^§^ |
| Age (median IQR, years) | 36.1 (26.6-49.0) | 36.7 (28.5-58.5) | 0.448* |
| Age of onset (median IQR, years) | 24 (19-38) | 25 (21-50) | 0.336* |
| Smoking (current/past/never), n (%) | 4/3/26 (12.1/9.1/78.8) | 1/4/19 (4.2/16.7/79.1) | 0.3716^§^ |
| Disease duration (median IQR, years) | 6.5 (3.6-13.6) | 6.4 (1.2-7.55) | 0.312* |
| Disease extent (pancolitis / left-sided), n (%) | 23/10 (69.7/30.3) | 17/7 (70.8/29.2) | 1.000^§^ |
| Steroid (naïve/dependent/refractory) , n (%) | 5/19/9 (15.2/57.6/27.3) | 2/12/10 (8.3/50.0/41.7) | 0.167^§^ |
| Biologics naïve, n (%) | 10 (30.3) | 5 (20.8) | 0.547^§^ |
| Lichtiger index (median IQR) | 7 (5-9) | 8 (8-10) | 0.029* |
| Mayo endoscopic subscore (median IQR) | 2 (2-3) | 2 (2-3) | 0.762* |
| Hb (median IQR, g/dL) | 12.8 (11.2-13.8) | 119 (10.6-13.6) | 0.290* |
| Alb (median IQR, g/dL) | 3.6 (3.5-4.0) | 3.6 (3.1-3.8) | 0.283* |
| CRP (median IQR, mg/dL) | 0.30 (0.08-1.61) | 0.89 (0.12-2.53) | 0.373* |
|  |  | *Mann-whitney U test, ^§^Fisher exact test. | |
| Alb; Albumin, CRP; C-reactive protein, Hb; Hemoglobin, IQR; interquartile range, | | | |

Supplementary table 2. Clinical remission week 8 for estimating clinical remission week 24.

|  |  | Clinical remission at week 24 | |
| --- | --- | --- | --- |
|  |  | (+) | (-) |
| Clinical remission at week 8 | (+) | 23 | 4 |
|  | (-) | 10 | 20 |

Supplementary table 3. Clinical remission week 16 for estimating clinical remission week 24.

|  |  | Clinical remission at week 24 | |
| --- | --- | --- | --- |
|  |  | (+) | (-) |
| Clinical remission at week 16 | (+) | 29 | 6 |
|  | (-) | 4 | 18 |
